# Comparison of translation algorithms in determining maximum allowable CTV shifts for Real‐Time Gated Proton Therapy (RGPT) robustness evaluation in prostate cancers

**DOI:** 10.1002/acm2.14543

**Published:** 2024-10-03

**Authors:** James Kuan Huei Lee, Kah Seng Lew, Calvin Wei Yang Koh, James Cheow Lei Lee, Andrew A. Bettiol, Sung Yong Park, Hong Qi Tan

**Affiliations:** ^1^ Department of Physics National University Singapore Singapore Singapore; ^2^ Division of Radiation Oncology National Cancer Centre Singapore Singapore Singapore; ^3^ Oncology Academic Clinical Programme Duke‐NUS Medical School Singapore Singapore

**Keywords:** fiducial markers, motion management, PBS, proton therapy, RGPT

## Abstract

**Introduction:**

Real‐Time Gated Proton Therapy (RGPT) is an active motion management technique that utilizes treatment gating and tumor tracking via fiducial markers. When performing RGPT treatment for prostate cancer, it is essential to account for the *CTV displacement relative to the body* in the clinical workflow. The workflow at the National Cancer Centre Singapore (NCCS) includes bone matching via CT‐CBCT images, followed by fiducial matching via pulsed fluoroscopy (soft tissue matching), and finally, a robustness evaluation procedure to determine if the difference is within an allowable tolerance. In this study, we compare two CTV translation methods for robustness evaluation: (1) an in‐house translation algorithm and (2) the RayStation “simulate organ motion” Deformable image registration (DIR) algorithm.

**Methods:**

Nine RGPT prostate patient plans with CTV volumes ranging from 17.1 to 96.72 cm^2^ were included in this study. An in‐house translation algorithm and “simulate organ motion” DIR RayStation algorithm were used to generate CTV shifts along R‐L, I‐S, and P‐A axes between ±10 mm at 2 mm steps. At each step, dose metrics, which include CTV D_max_, CTV D_95%_, and CTV D_98%_, were extracted and used as comparative metrics for CTV target coverage and hot spot evaluation.

**Results:**

Across all axes, there were no statistically significant differences between the two algorithms for all three dose metrics: CTV D_max_ (*P* = 0.92, *P* = 0.91, and *P* = 0.47), CTV D_95%_ (*P* = 0.97, *P* = 0.22, and *P* = 0.33), and CTV D_98%_ (*P* = 0.85, *P* = 0.33, and *P* = 0.36). Further, the in‐house translation algorithm evaluation time was less than 10 s, two orders of magnitude faster than the DIR algorithm.

**Conclusion:**

Our results demonstrate that the simpler in‐house algorithm performs equivalently to the realistic DIR algorithm when simulating CTV motion in prostate cancers. Furthermore, the in‐house algorithm completes the robustness evaluation two orders of magnitude faster than the DIR algorithm. This significant reduction in evaluation time is crucial especially when preparatory time efficiency is of paramount importance in a busy clinic.

## 1 INTRODUCTION

Proton therapy is recognized for its advantages over conventional x‐ray radiotherapy in terms of dose distributions and greater normal tissue‐sparing capabilities.^1^, ^2^, ^3^, ^4^, ^5^, ^6^ Nonetheless, the dose uncertainty induced by organ motion still remains a significant concern.^7^ Furthermore, in a pencil beam scanning (PBS) spot‐scanning proton beam delivery system, the proton beams are highly sensitive to organ motion. Anatomical changes in the proton beam path during a single fraction (intra‐fraction) and throughout the course of proton therapy (inter‐fraction) may substantially compromise the conformality and homogeneity of dose distributions within the tumor target and reduce the effectiveness of normal tissue sparing.^8^, ^9^, ^10^, ^11^ Motion‐induced dose uncertainties have been extensively studied for proton therapy, and several strategies have been proposed and implemented at various proton therapy centers. These strategies can be broadly classified into two categories^7^: (1) to prevent or reduce anatomical motion (active motion management), or (2) to account for organ motion during treatment planning or delivery (passive motion management). The former includes strategies such as breath holding (BH)^12^ or deep‐inspiration breath holding (DIBH),^13^ abdominal compression,^14^, ^15^ tumor tracking,^16^ and treatment gating^17^ while the latter includes strategies such as additional target margins, 4‐Dimensional (4D) treatment planning,^18^, ^19^, ^20^, ^21^, ^22^ and rescanning.^23^, ^24^, ^25^ A combination of two or more of these strategies can also be employed.^26^

Within the last decade, there has been considerable research into Real‐Time Gated Proton Therapy (RGPT),^27^, ^28^, ^29^, ^30^, ^31^, ^32^ a form of active motion management technique that gates the proton beam based on fiducial markers with pulsed fluoroscopy. It is co‐developed by Hokkaido University Hospital and Hitachi Ltd.^32^ At the National Cancer Centre Singapore (NCCS), we have employed Hitachi's RGPT system and have recently performed the acceptance testing (AT) and commissioning of the RGPT system. The commissioning workflow and daily and monthly quality assurance (QA) procedures of the RGPT system at NCCS have also been reported.^33^ Treatment for prostate and liver cancer patients with RGPT had also begun at NCCS in September 2023.

Recent studies have highlighted the substantial intra‐fractional motion of the prostate^34^, ^35^, ^36^ and its variability between patients.^36^, ^37^, ^38^, ^39^ As such, several immobilization techniques have been utilized in clinics for prostate treatment in proton therapy.^40^ Many clinics use rectal balloons inflated with either water or air, alongside maintaining a modestly full bladder, to immobilize the prostate and stabilize the rectum.^41^ More recently, biodegradable rectal spacers, such as hydrogel, have also been used in some clinics.^42^ These spacers are inserted between the prostate and rectum, displacing the anterior rectal wall from the prostate, thereby contributing to prostate immobilization. At our center, we employ SpaceOAR to reduce rectal radiation dose during treatment.^43^ Although the motion of the clinical target volume (CTV) in RGPT treatments is tracked using fiducial markers, excessive shifts can result in a significant displacement of the position of the CTV relative to the body. Consequently, the water equivalent thickness (WET) along the beam path will be affected, potentially compromising the dose coverage to the CTV. It is thus essential to account for the *CTV displacement relative to the body* (compared to the planning CT) in the clinical workflow during the RGPT treatment of prostate cancer. The RGPT clinical workflow for prostate cancer at NCCS includes bone matching via computed tomography cone‐beam computed tomography (CT‐CBCT) images, followed by fiducial matching via pulsed fluoroscopy (soft tissue matching), and finally a robustness evaluation to determine if the difference is within an allowable tolerance. Thus far, a robustness evaluation procedure for RGPT has not been reported or published before in past literature. In view of this, the main goal of this paper is to compare two CTV translation methods for the robustness evaluation step in the RGPT clinical workflow for prostate cancer: (1) an in‐house CTV translation algorithm, and (2) the RayStation “simulate organ motion” deformable image registration (DIR) based algorithm.^44^ The former is a more simplistic algorithm that only accounts for the CTV movement along the x, y, and z axes, while the latter is a more realistic representation of the CTV motion by means of deformation.

## 2 METHODS

### 2.1 NCCS proton therapy and RGPT

The NCCS Hitachi Probeat proton therapy system commissioned in 2022 utilizes the PBS spot‐scanning delivery technique with a synchrotron accelerator. It can deliver 98 discrete energy layers ranging from 70.2 to 228.7 MeV. NCCS has four fully rotating gantries, each equipped with two pairs of x‐ray sources and flat panel detectors (FPDs). All gantries are capable of performing RGPT.

**FIGURE 1:**
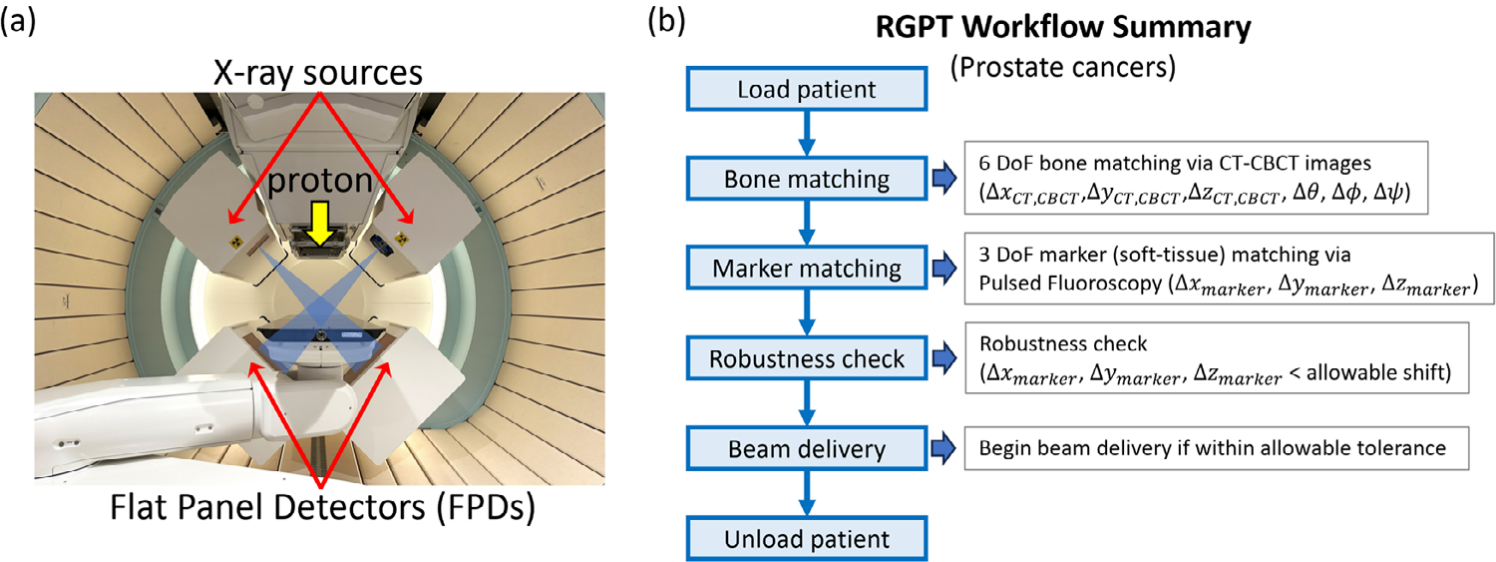
(a) The NCCS RGPT system. (b) A schematic of the clinical workflow at NCCS for RGPT treatment of prostate cancers.

Figure 1a shows the RGPT setup at NCCS: two orthogonal sets of extendable x‐ray sources and FPDs installed in the gantry of the system are used to observe implanted fiducial markers and bony structures during kV‐kV, CBCT, and RGPT fluoroscopic imaging. During RGPT, the proton beam is gated when the fiducial marker enters a predefined gating window. This is monitored via real‐time images of the fiducial markers implanted near or adjacent to the CTV via RGPT fluoroscopic imaging such that any motion attributed to the tumor can be accurately tracked in real‐time via the fiducial marker positions that are displayed on the RGPT graphical user interface (GUI).

### 2.2 RGPT clinical workflow for prostate cancer

Figure 1b shows a schematic of the RGPT clinical workflow for the treatment of prostate cancer at NCCS. The workflow can be segmented into three parts: (1) a bone matching via CT‐CBCT images, (2) a fiducial marker matching via pulsed fluoroscopy (soft tissue matching), and finally (3) a robustness evaluation procedure to determine if the difference is within an allowable tolerance. In the first part, CT and CBCT images of the patient are acquired. Six degrees of freedom (DoF) $\left(\Delta x_{CT,CBCT},\Delta y_{CT,CBCT},\Delta z_{CT,CBCT},\Delta \theta ,\Delta \phi ,\Delta \psi \right)$ bone matching is then performed, where the bone structures in the CT images are aligned with the bone structures in the CBCT images. In the second part, fluoroscopic images of the patient are acquired via pulsed fluoroscopy. Three DoF $\left(\Delta x_{marker},\Delta y_{marker},\Delta z_{marker}\right)$ fiducial marker matching is then performed. The final part involves performing a robustness evaluation to determine if the difference in the fiducial matching is within an acceptable tolerance level ($\Delta x_{marker},\Delta y_{marker},\Delta z_{marker}<allowable\;shifts$), defined by the allowable marker shifts. At NCCS, we adopt a clinical goal of CTV D_95% _> 95% of the dose prescription, where the CTV D_95%_ dose metric is used to evaluate the amount of dose delivered to the CTV. An in‐house algorithm is used to calculate the CTV D_95%_ values at specific user‐defined marker shifts. The maximum allowable shifts in the right‐left (R‐L), inferior‐superior (I‐S), and posterior‐anterior (P‐A) directions are then determined by comparing these values to the clinical goal. If the observed marker shift during treatment exceeds this tolerance threshold, troubleshooting is required to identify whether the issue is due to marker tracking loss or actual marker deviation. Depending on the issue, treatment may need to be paused or stopped until rectified.

### 2.3 Patient data and characteristics

Nine patient plans that were treated with RGPT for prostate cancer were used in this retrospective study for robustness evaluation simulations. The treatment plans were robustly optimized with a 3.5% range and 5 mm setup uncertainty. Table 1 summarizes the beam angles and prostate CTV volumes for the patient plans examined in this study. The CTV volumes for these patients ranged from 17.1 to 96.72 cm^2^ and gold Anchor internal fiducial markers each measuring 0.4 × 10.0 mm^2^ was used during the RGPT treatments. Notably, patients 1 to 5 were treated using two beam angles while patients 6 to 9 were treated using four beam angles. This was due to patients 6 to 9 having cancer affecting both the prostate and lymph nodes.

**TABLE 1:** Summary of prostate CTV volumes and beam angles for all nine patients treated with RGPT for prostate cancer.

	P1	P2	P3	P4	P5	P6	P7	P8	P9
**Target volume (cc)**	41.9	95.6	96.7	50.1	42.0	34.0	17.1	32.2	58.1
**Beam 1 (deg)**	265.0	265.0	265.0	265.0	270.0	270.0	265.0	270.0	260.0
**Beam 2 (deg)**	95.0	95.0	90.0	93.0	90.0	90.0	90.0	90.0	95.0
**Beam 3 (deg)**	–	–	–	–	–	200.0	210.0	210.0	210.0
**Beam 4 (deg)**	–	–	–	–	–	160.0	150.0	150.0	150.0

### 2.4 The in‐house translation algorithm

An in‐house translation algorithm was developed using the scripting environment within RayStation 2023b (RaySearch Laboratories, Stockholm, Sweden) to generate CTV shifts along the x, y, and z axes (R‐L, I‐S, and P‐A axes) for each prostate patient plan. The algorithm uniformly translates the voxels of the CTV Region of Interest (ROI) without rotation, scaling, or deformation. The ROI transformation process in RayStation is executed through two main steps: (1) the CTV ROI contour is transformed using the transformation matrix M, and (2) the point of interest (POI) isocenter is shifted to re‐initialize the isocenter following the translation. The transformation matrix M and the adjustment of the POI isocenter are described as follows in RayStation:

(1) $$M=\left(\begin{array}{cccc} 1 & 0 & 0 & x\\ 0 & 1 & 0 & y\\ 0 & 0 & 1 & z\\ 0 & 0 & 0 & 1 \end{array}\right)$$

(2) $$\text{New POI Isocenter}=\left(x_{0}+x,y_{0}+y,z_{0}+z\right)$$

where $\left(x_{0},y_{0},z_{0}\right)$ are the co‐ordinates of the original POI isocenter localized at the initial axial view position with x,y,andz representing the user‐defined CTV shift magnitudes in cm along the R‐L, I‐S, and P‐A axes, respectively. For example, a shift of +4 mm solely in the x‐direction would mean setting x=0.4 and y=z=0.

For robustness evaluation, a total of 30 CTV shifts (10 shift points per axis) were generated with this algorithm for each prostate patient plan. These shifts ranged from −10 to 10 mm in 2 mm increments along the x, y, and z axes. At each shift magnitude, dose metrics including CTV D_max_, CTV D_95%_, and CTV D_98%_ were computed and extracted. Additionally, dose metrics for the nominal scenario (no translation) were also extracted.

### 2.5 The “simulate organ motion” DIR‐based algorithm

The “simulate organ motion” tool in the RayStation treatment planning system (TPS) utilizes a DIR algorithm based on the ANAtomically CONstrained Deformation Algorithm (ANACONDA).^45^ The ANACONDA DIR algorithm works by creating a deformable registration between the original and new ROI positions while deforming other ROIs and CT values accordingly. The DIR problem is formulated as a non‐linear optimization problem, with the objective function comprising a weighted sum of four non‐linear terms: image similarity, grid regularization, shape‐based regularization, and a penalty term.

On the other hand, the “simulate organ motion” DIR‐based tool enhances the ANACONDA algorithm by allowing users to specify the motion ROI (typically the CTV) and the fixed ROIs (specified as non‐CTV structures deemed immobile). During the deformation process, the user‐defined motion magnitude of the motion ROI drives the deformation of the patient anatomy, while the fixed ROIs remain static. Structures that are neither motion nor fixed ROIs are deformably mapped and are perceived to move together with the motion ROI.

The “simulate organ motion” DIR‐based tool can be used for robustness evaluation or as input for planning robustly against intra‐fractional or inter‐fractional organ motion.^44^ In this study, we used the DIR‐based tool solely for the robustness evaluation of the prostate CTV intra‐fractional motion, designating the prostate CTV as the motion ROI and the left and right femoral heads as the fixed ROIs.

Similarly, for robustness evaluation, a total of 30 deformed images (10 deformed images per axis) were generated with this algorithm for each prostate patient plan. These shifts ranged from −10 to 10 mm in 2 mm increments along the x, y, and z axes. At each shift magnitude, dose metrics including CTV D_max_, CTV D_95%_, and CTV D_98%_ were computed and extracted. Additionally, dose metrics for the nominal scenario (no deformation) were also extracted.

### 2.6 Comparison of algorithms to calculate allowable marker shifts

Both algorithms operate under the assumption that the bone‐matching step in the RGPT clinical workflow has been satisfactorily completed and that an acceptable level of bone matching has already been achieved. The in‐house translation and the “simulate organ motion” DIR RayStation algorithms were utilized to generate CTV shifts along the x, y, and z axes, ranging from −10 to 10 mm in 2 mm increments. At each increment, dose metrics, including CTV D_max_, CTV D_95%_, and CTV D_98%_, were extracted from each algorithm and served as comparative metrics for CTV target coverage and hot spot evaluation. The dose delivered at x = y = z = 0 (the nominal scenario) is referred to as the nominal dose, representing a 100% dose prescription. Percentage deviations were calculated for each patient, for each dose metric, and at each shift point by taking the difference between the calculated dose value and the nominal dose value, dividing it by the nominal dose value, and then normalizing it to 100%. This process was applied to both algorithms. The resulting value indicates the percentage deviation from the 100% dose prescription at each shift point. As previously mentioned, NCCS adopts a clinical goal of achieving CTV D_95%_ > 95% of the dose prescription. A percentage deviation of less than 5% for the CTV D_95%_ dose metric indicates that the clinical goal has been met for that shift point, and vice versa. The maximum allowable shifts in the R‐L, I‐S, and P‐A directions can then be determined by identifying the maximum shift values corresponding to a percentage deviation of less than 5%.

Both algorithms used the RayStation 2023b Monte Carlo (MC) based ‘Ion Monte Carlo’ dose engine for dose calculations. Nine Kruskal–Wallis non‐parametric tests were performed for the dose metrics (CTV D_max_, CTV D_95%_, and CTV D_98%_) along each axis (x, y, and z) to compare the doses extracted from the two different translation algorithms. A two‐tailed test of *P*‐values of less than 0.05 was considered statistically significant.

## 3 RESULTS

### 3.1 Comparison of dose metrics extracted from algorithms

**FIGURE 2:**
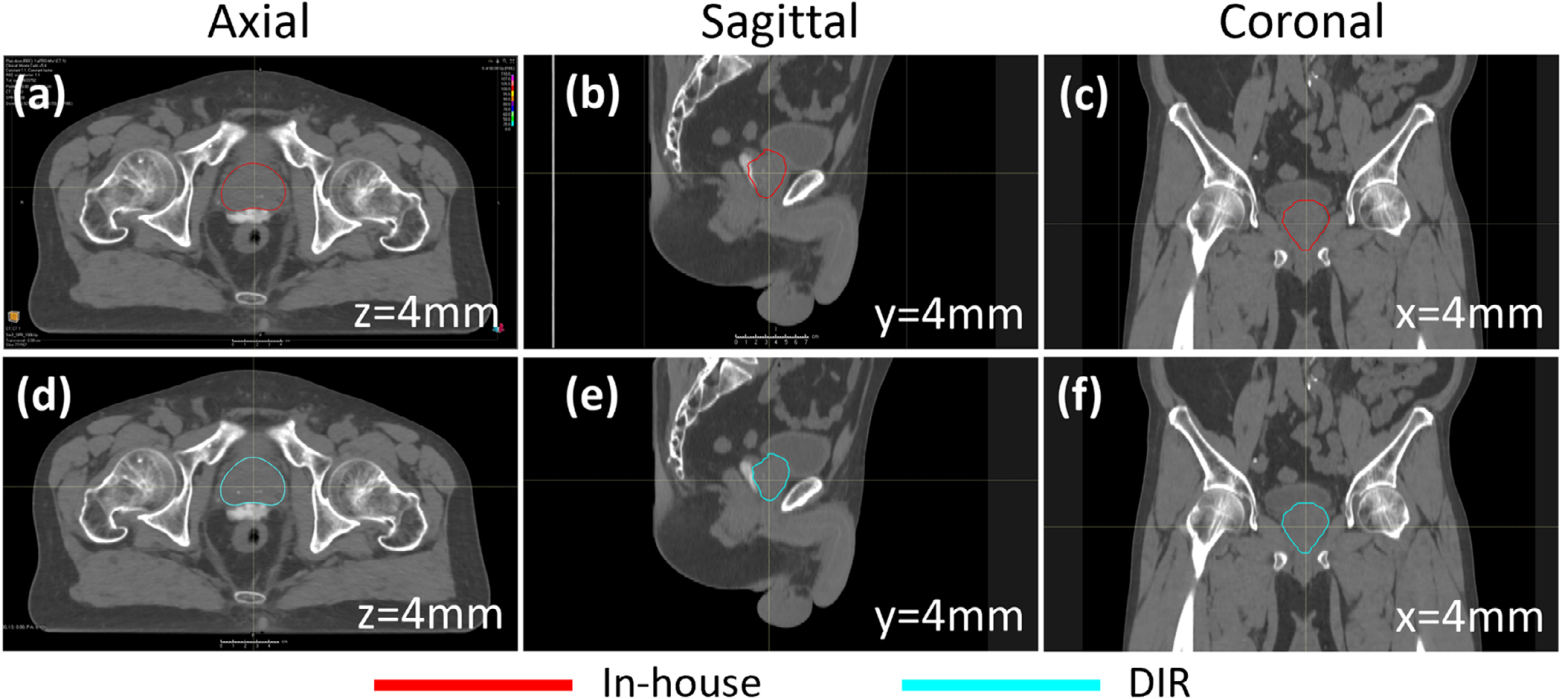
Side‐by‐side computed tomography (CT) image comparisons of the CTV shifts via the in‐house translation algorithm (red contour) and the “simulate organ motion” DIR algorithm (blue contour) for a shift of +4 mm along the (a, d) z, (b, e) y, and (c, f) x‐axis as depicted in the axial, sagittal and coronal planes respectively, for patient 4.

**FIGURE 3:**
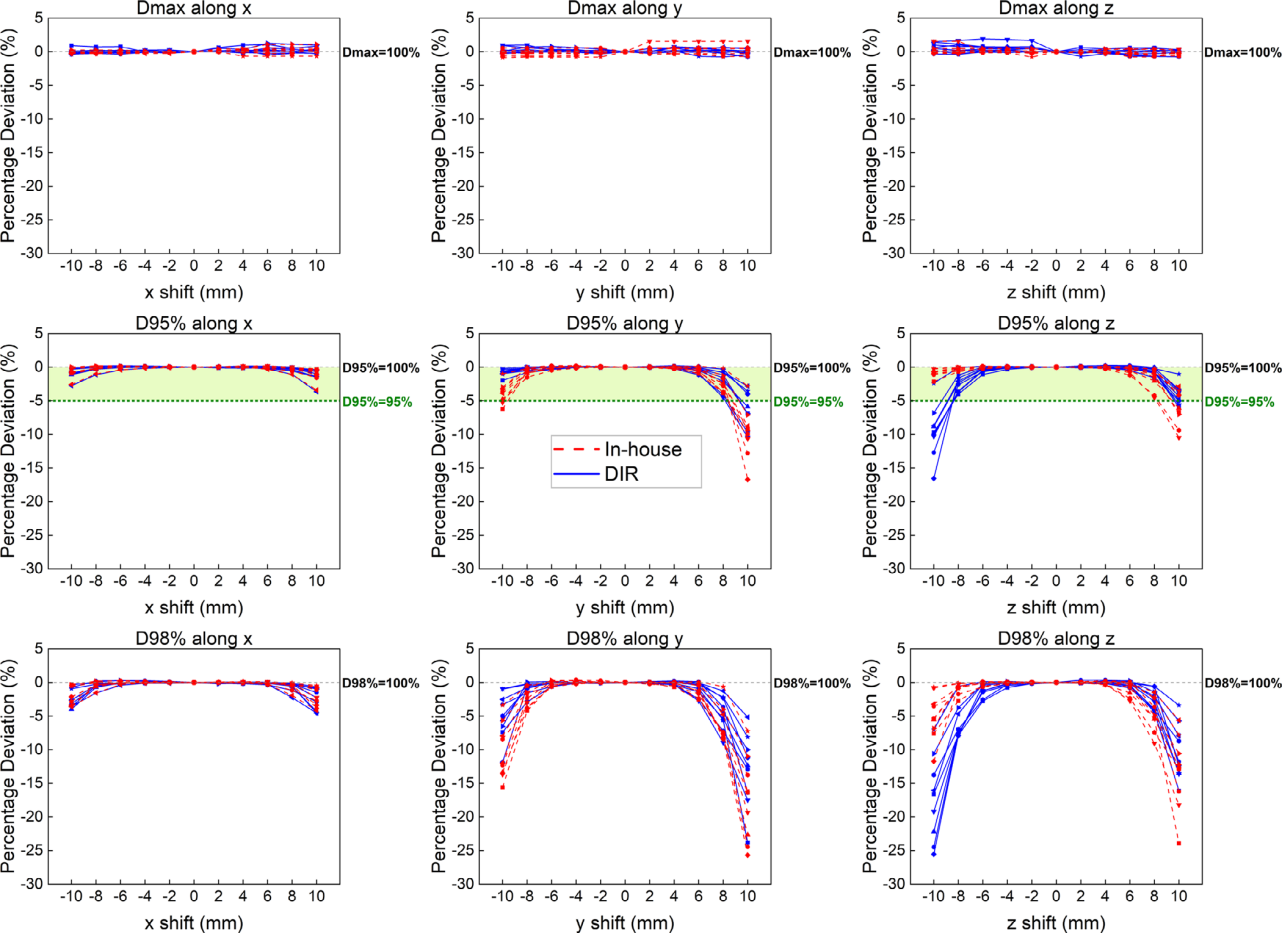
Percentage deviations for dose metrics CTV D_max_, CTV D_95%_, and CTV D_98%_ for nine patients via the in‐house translation algorithm (red) and the “simulate organ motion” DIR algorithm (blue). D_X_ = 100% indicates that 100% of the dose prescription has been reached, where X denotes the respective dose metric. D_95%_ = 95% is also shown for CTV D_95%_, following the clinical criteria of CTV D_95%_ > 95% dose prescription used at NCCS.

**FIGURE 4:**
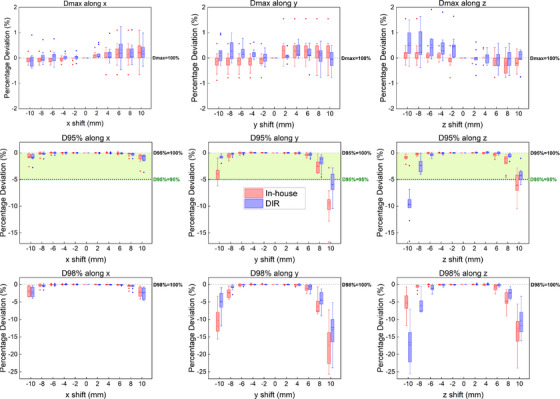
Aggregate data represented by box and whisker plots for dose metrics CTV D_max_, CTV D_95%_, and CTV D_98%_ for nine patients via the in‐house translation algorithm (red) and the “simulate organ motion” DIR algorithm (blue). Squares indicate the mean while the diamonds indicate outliers. D_X_ = 100% indicates that 100% of the dose prescription has been reached, where X denotes the respective dose metric. D_95%_ = 95% is also shown for CTV D_95%_, following the clinical criteria of CTV D_95%_ > 95% dose prescription used at NCCS.

Figure 2 shows side‐by‐side CT images comparing CTV shifts by each algorithm for one patient (P4) along the axial, sagittal, and coronal planes. The CT images show the contours of the translated CTVs at shifts of 4 mm along the z, y, and x axes respectively. Figure 3 shows the percentage deviation from the 100% dose prescription for dose metrics CTV D_max_, CTV D_95%_, and CTV D_98%_ along the x, y, and z axes for both translation algorithms. Figure 4 represents the aggregate data in the form of box and whisker plots. Along the x‐axis, both algorithms performed very similarly across all dose metrics (CTV D_max_, *P* = 0.92; CTV D_95%_, *P* = 0.97; CTV D_98%_, *P* = 0.85). Further, both algorithms reflected similar patient‐specific characteristics. For example, along the x‐axis for CTV D_95%_, the largest percentage deviation occurred in patient 6 at x = 10 mm for both algorithms (in‐house: −3.41% vs. DIR: −3.64%) followed by at x = −10 mm (in‐house: −2.59% vs. DIR: −2.76%). Similarly, along the x‐axis for CTV D_98%_, patient 6 again demonstrated the largest percentage deviation across both algorithms at x = 10 mm (in‐house: −4.17% vs. DIR: −4.53%) followed by at x = −10 mm (in‐house: −3.56% vs. DIR: −3.54%). Along the y and z axis for all three dose metrics, no statistical differences were observed between the algorithms (CTV D_max_, *P* = 0.91 and *P* = 0.47; CTV D_95%_, *P* = 0.22 and *P* = 0.33; CTV D_98%_, *P* = 0.33 and *P* = 0.36, respectively).

However, visual differences begin to emerge at the larger shift points of ±8 and ±10 mm. For example, for CTV D_95%_ along the y‐axis, the largest *divergence* between algorithms (difference between the percentage deviations) occurred in patient 5 at y = 10 mm, with the in‐house algorithm showing a percentage deviation of −16.7 % while the DIR algorithm exhibited a deviation of only −4.00 %. Similarly, for CTV D_95%_ along the z‐axis, the largest divergence was observed in patient 5 but at z = −10 mm, with the in‐house algorithm showing a deviation of −0.660% compared to −16.6% for the DIR algorithm. For CTV D_98%_ along the y‐axis, the largest divergence was again observed in patient 5 at y = 10 mm, with the in‐house algorithm showing a deviation of −25.7%, whereas the DIR algorithm showed a deviation of −11.2%. For CTV D_98%_ along the z‐axis, the largest divergence was found in patient 2 at z = −10 mm, with the in‐house algorithm showing a deviation of −3.55% compared to −24.5% for the DIR algorithm. These are reflected in Table 2, along with the median dose values for each algorithm across each axis and their corresponding *P*‐values.

**TABLE 2:** Median dose values for each algorithm along each axis, with the corresponding *P*‐values from the Kruskal–Wallis test. The worst‐case scenario (the largest difference between the percentage deviations of the algorithms) for each axis, along with the shift value at which it occurs, is also shown.

			Median dose value			Worst‐case scenario		
Target	Dose metric	Axis	In‐house (Gy)	DIR (Gy)	*P*‐value	In‐house (%)	DIR (%)	Shift value (mm)
CTV	D_max_	x	64.21	64.06	0.92	0.56	−0.32	10
		y	64.21	64.13	0.91	−0.16	0.97	−10
		z	64.21	64.15	0.47	−0.28	1.40	−10
	D_95%_	x	60.19	60.23	0.97	−3.41	−3.64	10
		y	59.77	59.88	0.22	−16.7	−4.00	10
		z	59.88	59.80	0.33	−0.66	−16.6	−10
	D_98%_	x	59.72	59.70	0.85	−4.17	−4.53	10
		y	59.44	59.47	0.33	−25.7	−11.2	10
		z	59.47	59.45	0.36	−3.55	−24.5	−10

The subsequent nine Kruskal–Wallis tests did not reject the null hypothesis, with low H values ranging from 0.0095 to 1.5 and high *P*‐values ranging from 0.22 to 0.97. These results attest that there are no statistically significant differences between the doses extracted from the two translation algorithms for any of the dose metrics (CTV D_max_, CTV D_95%_, and CTV D_98%_) across any of the axes (x, y, and z). The high *P*‐values suggest that the performance of the two algorithms is comparable for all metrics and axes evaluated in this study.

### 3.2 Maximum allowable shifts based on NCCS clinical goals

At NCCS, we adopt a clinical goal of achieving CTV D_95%_ > 95% of dose prescription for RGPT treatment of prostate cancers. Following this benchmark, the maximum acceptable percentage deviation for both the in‐house and DIR algorithm is 5% for CTV D_95%_. For reference, this clinical goal is illustrated in green for the CTV D_95%_ dose metric in Figures 3 and 4.

For CTV D_95%_ along the x‐axis, both algorithms did not exceed this 5% percentage deviation (< ±2%) for the entire range of −10 to 10 mm. The largest percentage deviation was only −3.41% for the in‐house algorithm and −3.64% for the DIR algorithm, as previously mentioned. For CTV D_95%_ along the y‐axis, the in‐house translation algorithm exceeded the 5% percentage deviation at shift points y = −10 mm and y = 10 mm. Therefore, the maximum allowable shifts for the in‐house translation algorithm were determined to be −8 to 8 mm. In contrast, the DIR algorithm only exceeded the 5% percentage deviation at y = 10 mm, making the maximum allowable shifts for the DIR algorithm −10 to 8 mm. For CTV D_95%_ along the z‐axis, the in‐house algorithm exceeded the acceptable deviation at z = 10 mm, while the DIR algorithm exceeded the acceptable deviation at both z = −10 and 10 mm. Consequently, the maximum allowable shifts for the in‐house translation algorithm were determined to be −10 to 8 mm, while for the DIR algorithm, the range was determined to be −8 to 8 mm.

### 3.3 Algorithm evaluation times

**FIGURE 5:**
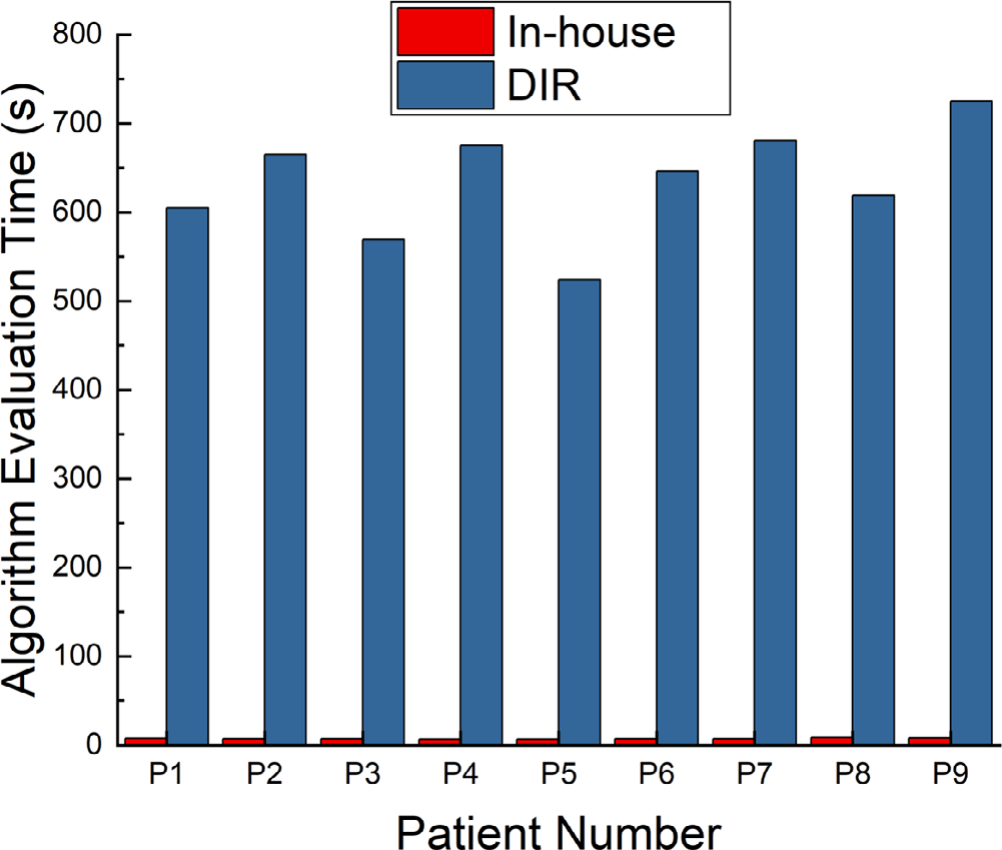
The total evaluation time taken for the in‐house translation algorithm (red) and the “simulate organ motion” DIR algorithm for all 30 (x, y, and z) shifts/deformations, per patient.

Figure 5 shows the evaluation time for generating a total of 30 shifts (10 for each axis) per patient, for each algorithm. To measure evaluation times, we utilized the timing functions available in the Python scripting environment. The in‐house translation algorithm evaluation time ranged from 6.56 s (P4) to 8.44 s (P8), while the DIR algorithm ranged from 604.83 s (P1) to 725.18 s (P9). Note that the evaluation time does not include dose calculation time to highlight the differences solely due to the different translation methods.

## 4 DISCUSSION

In this study, we compared and detailed two CTV translation algorithms for the robustness evaluation step for RGPT treatment of prostate cancers. The key difference between the two algorithms is in the way the CTV is transformed. The in‐house algorithm accounts only for the translation of the CTV along the x, y, and z axes, without incorporating rotation, scaling, or deformation, whereas the “simulate organ motion” DIR algorithm considers CTV motion through deformation. The DIR algorithm, although realistic, requires a considerably longer robustness evaluation time as compared to the in‐house translation algorithm (Figure 5). The longer evaluation time associated with the “simulate organ motion” algorithm, which is based on the DIR technique, can be attributed to the creation of new deformed CT examination objects in RayStation, as well as the intrinsic complexity of the deformation process. Notably, the evaluation time for the in‐house translation algorithm is two orders of magnitude faster than the DIR algorithm.

We evaluated the performance of both algorithms on how similarly they fared using dose metrics typically used for target coverage and hot spot evaluation. The CTV D_max_ dose metric was used for hot spot evaluation, while the CTV D_95%_ and D_98%_ dose metrics were used for target coverage evaluation (Figures 3 and 4). There were no statistical differences between the doses extracted from the algorithms for all dose metrics across all axes.

Both algorithms demonstrated improved robustness towards intra‐fractional CTV motion along the x‐axis for CTV D_max_, CTV D_95%_, and CTV D_98%_, as compared to the y or z axes. This is expected since most of the beam weight in RGPT prostate planning is optimized along the R‐L direction (x‐axis). Therefore, when the CTV is shifted laterally, we would expect the algorithms to reflect minimal percentage deviations along the x‐axis, even at extreme shift points, as opposed to shifts along other axes. For CTV D_max_, the percentage deviations for both algorithms remained very minimal (< ± 2%) across the entire shift range for all axes. In fact, for the dose metrics along the x‐axis, both algorithms captured and reflected similar characteristics from the patient data.

Conversely, an underdose becomes more apparent at larger shift points of ± 8 and ± 10 mm along the y and z axes for target coverage dose metrics CTV D_95%_ and D_98%_, which is anticipated when the CTV begins to shift away from these field directions. For CTV D_95%_ and D_98%_ along the y and z axes, there were no statistical differences between the doses extracted from the algorithms, although differences between the algorithms become more noticeable at extreme shift points of ± 8 and ± 10 mm. For example, the largest divergence between the algorithms occurred at CTV D_98%_ at shift point z = −10 mm.

Adhering to the clinical goal of maintaining CTV D_95%_ > 95% of the dose prescription, ± 8 mm are the maximum allowable shift distances for CTV D_95%_ along the y and z axes where both algorithms agree on percentage deviations of less than 5% (i.e., CTV D_95%_ > 95% dose prescription). Although the in‐house and DIR algorithms slightly differ in their determination of the maximum allowable shifts along the y and z axes, neither algorithm consistently overestimates this maximum shift range. For instance, for CTV D_95%_ along the y‐axis, the in‐house algorithm sets a stricter maximum allowable shift range of y = −8 to 8 mm, whereas for CTV D_95%_ along the z‐axis, the DIR algorithm imposes the stricter maximum allowable shift range of z = −8 to 8 mm.

Although the RGPT patient plans used in this retrospective study included patients with node‐positive prostate cancer, the CTV motion simulation algorithms could also be extended to patients with prostate cancer in general as there were no marked differences in CTV motion between those with prostate cancer and those with node‐positive prostate cancer.

It is important to note that this study was conducted exclusively on prostate cases. For more complex and mobile anatomies, the DIR algorithm may offer greater accuracy than the in‐house translation. For instance, in liver cases, the magnitude of CTV shifts is significantly larger. Using the in‐house translation algorithm in these scenarios could result in the CTV potentially occupying the inferior section of the lung. Therefore, caution is advised when extending the use of the in‐house translation algorithm beyond prostate cases.

Some centers use alternative techniques for real‐time tumor tracking such as using radio frequency (RF) emitting implants (e.g., Calypso^46^). In such cases, the in‐house algorithm retains its capability to simulate CTV motion regardless of whether RF emitting implants or implanted gold fiducial markers are used, as the in‐house algorithm requires only the specification of the CTV ROI of the tumor target as input, along with the re‐initialization of the POI isocenter following the translation of the CTV ROI.

The “simulate organ motion” DIR algorithm is a feature that is unique to the RayStation TPS. As more Hitachi particle therapy centers adopt RGPT as their motion management solution, centers using different treatment planning systems may find our in‐house algorithm a practical alternative for simulating CTV motion during the robustness evaluation step of their RGPT clinical workflow, or for prostate CTV motion simulations in general.

## 5 CONCLUSION

This study compared two methods for CTV translation in the robustness evaluation step of the RGPT clinical workflow for prostate cancer: an in‐house algorithm and the “simulate organ motion” DIR algorithm. Our results demonstrate that the simpler in‐house algorithm performs equivalently to the more realistic DIR algorithm when simulating CTV motion in prostate cancers. Further, the in‐house algorithm completes the robustness evaluation two orders of magnitude faster than the DIR algorithm. This significant reduction in evaluation time makes the in‐house algorithm a preferred choice for robustness evaluation for a busy clinic, where preparatory time efficiency is of paramount importance. Moreover, the “simulate organ motion” DIR algorithm is a feature unique to the RayStation TPS. As such, centers using different treatment planning systems could find our in‐house algorithm to be a practical alternative for simulating CTV motion in prostate cancers.

## AUTHOR CONTRIBUTIONS

*Study conception and design*: Hong Qi Tan. *Data acquisition and analysis*: James Kuan Huei Lee. *Data interpretation*: All authors. *Statistical analyses*: James Kuan Huei Lee and Hong Qi Tan. *Obtained funding*: Hong Qi Tan. *Administrative, technical, or material support*: Hong Qi Tan. *Study supervision*: Andrew A. Bettiol, Hong Qi Tan, and Sung Yong Park. *Drafting of the manuscript*: James Kuan Huei Lee. *Approval of final manuscript*: All authors.

## CONFLICT OF INTEREST STATEMENT

The authors declare no conflicts of interest.
